# Identification of Biomarkers Related to CD8^+^ T Cell Infiltration With Gene Co-expression Network in Lung Squamous Cell Carcinoma

**DOI:** 10.3389/fcell.2021.606106

**Published:** 2021-03-18

**Authors:** Min Tang, Yukun Li, Xianyu Luo, Jiao Xiao, Juan Wang, Xin Zeng, Qihao Hu, Xiaoyan Chen, Si-jie Tan, Jun Hu

**Affiliations:** ^1^Department of Cardiothoracic Surgery, The Second Affiliated Hospital, University of South China, Hengyang, China; ^2^The Second Affiliated Hospital, University of South China, Hengyang, China; ^3^Department of Histology and Embryology, Clinical Anatomy and Reproductive Medicine Application Institute, University of South China, Hengyang, China; ^4^Medical College, Hunan Polytechnic of Environment and Biology, Hengyang, China; ^5^Department of Endocrinology, The Affiliated Nanhua Hospital, University of South China, Hengyang, China

**Keywords:** biomarkers, CD8^+^ T cell, network, lung squamous cell carcinoma, METTL8, bioinformatics

## Abstract

Lung squamous cell carcinoma (LSCC) is one of the most common types of lung cancer in adults worldwide. With the development of modern medicine, cancer treatment that harnesses the power of the immune system might be particularly effective for treating LSCC. In this research, LSCC expression data, which quantify the cellular composition of immune cells, were analyzed by weighted gene coexpression network analysis (WGCNA) and a deconvolution algorithm based on the Gene Expression Omnibus (GEO) database, and the results indicated a close relationship between LSCC and CD8^+^ T cells. Six hub genes (SYT3, METTL8, HSPB3, GFM1, ERLIN2, and CLCN2) were verified by gene–gene network and protein–protein interaction (PPI) network analyses. We found that the six hub genes were increased in cancer tissues and were closely correlated with cancer development and progression. After immune correlation analysis, METTL8 was selected as a prognostic biomarker. Finally, we found that the METTL8 levels were increased in multiple lung cancer cell lines and LSCC tissues. METTL8 inhibition could clearly induce G1 cell cycle arrest and suppress proliferation. Therefore, METTL8, which is related to CD8^+^ T cell infiltration, might be identified as a potential biomarker and gene therapy target in LSCC.

## Introduction

Lung cancer is the most common etiology of human respiratory system cancer diagnoses worldwide. Annually, nearly 2.1 million new cases of lung cancer are diagnosed, and more than 1.8 million lung cancer-related deaths occur, accounting for approximately 18.4% of all oncology-related deaths (Siegel et al., 2018). Non-small cell lung cancer (NSCLC) is the most common type of lung carcinoma (Molina et al., 2008). Lung squamous cell carcinoma (LSCC) and lung adenocarcinoma (LUAD) have been considered different diseases since they originate from different cells and regions of the lung, present different features or states, and harbor various genetic alterations and epigenetic modifications. These differences suggest the need for different therapeutic strategies for lung cancer patients (Campbell et al., 2016). Therefore, exploring the pathophysiological mechanisms of different subtypes of lung cancer is helpful to realize accurate treatment and improve the survival rate of patients.

Recently, immune checkpoint inhibitors, such as inhibitors of programmed cell death 1 (PD-1) and its ligand (PD-L1), have become the first-line treatment for LSCC. To date, second-line drugs, such as nivolumab, pembrolizumab, and atezolizumab, are being used to treat LSCC (Julie et al., 2015; Fehrenbacher et al., 2016; Herbst et al., 2016; Luis et al., 2018). However, effective immunotherapy biomarkers still do not meet the needs of clinical diagnosis and treatment. Therefore, the study of immune-related molecular markers is an important focus of LSCC.

The effects of immunotherapy are easily affected by the tumor microenvironment in LSCC, especially by immune infiltration (Jiang et al., 2019). CD8^+^ T cells are the most important regulators of cancer adaptive immunity and mediate antitumor immunity by directly killing cancer cells (Daniel and Ira, 2013). Many immunosuppressive cells, such as regulatory B cells and tumor-associated macrophages, could impede the activation of CD8^+^ T cells to accelerate the formation, development, and progression of LSCC by regulating the levels of PD1 and PDL1 (Seo et al., 2018). Many studies have reported that immune-related genes (IRGs) play an important role in the early diagnosis and prognosis of LSCC, but these findings do not directly translate well into clinical applications (Trojan et al., 2004; Guo et al., 2018; Stankovic et al., 2018). A previous study indicated that the NSCLC microenvironment plays a key role in carcinogenesis *via* the infiltration of CD8^+^ FOXP3^+^ T cells, CD8^+^ T cells, and FOXP3^+^ T cells (Hao et al., 2020). PD-1 inhibition activates CD8^+^ T cells to increase T cell immunity, which induces cancer regression (Sui et al., 2018). Therefore, the activation of CD8^+^ T cells may be key to treating LSCC by immunotherapy (Daniel and Ira, 2013). Another study also found that the combination of oxymatrine and cisplatin could synergistically activate the anticancer CD8^+^ T cell immunity to treat cancer patients (Ye et al., 2018). Hence, the validation of hub IRGs associated with CD8^+^ T cell infiltration will help to monitor the immunotherapy response of LSCC and study the mechanism of immune infiltration.

However, using traditional molecular biological methods to explore immune-related biomarkers is complex and arduous (Guo et al., 2018). With the rapid development of bioinformatics, many tools have been used to search for biomarkers, especially immune-related biomarkers (Lin et al., 2020). To identify the hub immune-related biomarkers in LSCC, we first used weighted gene coexpression network analysis (WGCNA) (Langfelder and Horvath, 2008) to analyze LSCC gene level data. The estimating relative subsets ff RNA transcripts (CIBERSORT) algorithm (Chen et al., 2018) was utilized to analyze the immune cell compositions in LSCC samples (Li et al., 2020). Subsequently, the content of immune cells in each patient was used as the characteristic input, the WGCNA network was constructed together with the mRNA expression data to find the module genes most related to immune infiltration, and the specific molecular mechanism was further explored. Finally, prognostic immune-related biomarkers were validated. This is the first study to identify CD8^+^ T cell-related biomarkers in LSCC by WGCNA.

## Materials and Methods

### Gene Expression Data and Subsequent Processing Based on TCGA Database

TCGA database^1^ is the largest cancer gene information database and includes gene expression data, miRNA expression data and copy number variation, DNA methylation, SNPS, and other data. We downloaded the LSCC primitive mRNA expression processed data and collected 490 specimens (Blum et al., 2018).

### Weighted Gene Coexpression Network Analysis

The data File of Series Matrix File of GSE17710 (Wilkerson et al., 2010) was downloaded from the NCBI GEO public database^2^. The transcriptional data of 56 groups of LSCC patients were extracted for the construction of a WGCNA coexpression network to explore the differences in the molecular mechanisms of lung cancer progression. In this study, a weighted gene coexpression network was constructed to identify the gene module of coexpression and to explore the association between the gene network and phenotype as well as the core genes in the network. The WGCNA-R packet was used to construct the coexpression network of all the genes in the GSE17710 dataset. The genes with the first 5,000 variances were identified by this algorithm for further analysis, and the soft threshold was set to five. The weighted adjacency matrix was transformed into a topological overlap matrix (TOM) to estimate network connectivity, and the hierarchical clustering method was used to construct the cluster tree structure of the TOM matrix. Different branches of the cluster tree represent different gene modules, and different colors represent different modules. Based on the weighted correlation coefficient of genes, genes were classified according to their expression patterns: genes with similar patterns were grouped into one module, and genes were divided into several modules through their expression patterns (Langfelder and Horvath, 2008).

### Analysis of Immune Cell Infiltration

CIBERSORT was used to analyze the RNA-seq data from LSCC patients to determine the relative proportions of 22 types of immune-infiltrating cells. The content of immune cells in each patient was used as the characteristic input, the WGCNA network was constructed together with the mRNA expression data to identify the module genes most related to immune infiltration, and the specific molecular mechanism was further explored (Chen et al., 2018).

### Functional Enrichment Analysis of Gene Modules

Due to our interest in the WGCNA module (the research for the brown module and phenotypic correlation highest) function and signaling pathways involved in biology, the Metascape database^3^ was used for annotation and visualization of a specific gene module in Gene Ontology (GO) analysis and Kyoto Encyclopedia (KEGG pathway gene genome) analysis. Min overlap ≥ 3 and *P* ≤ 0.01 were considered to be statistically significant (Zhou et al., 2019).

### The Relationship Between Hub Genes and Immune Cells

The TIMER database^4^ is used to assess RNA-Seq expression spectrum data of immune cells infiltrating tumor tissues. In this study, the relationship between the hub gene and immune cell content was explored by using the TIMER database, and the inter-tumor infiltration level of changes in different somatic cell copy number of hub genes was compared (Pan et al., 2019).

### Gene Set Enrichment Analysis

Gene Set Enrichment Analysis (GSEA) uses a predefined gene set (based on the KEGG database) to sequence genes according to their differential expression levels in the two types of samples and then tests whether the preset gene set is enriched at the top or bottom of the sequencing table. In this study, GSEA was used to compare the signaling pathway differences between the high and low expression groups of core genes, to explore the possible molecular mechanisms of the difference in prognosis between the two groups and to provide molecular mechanism clues for the involvement of core genes in tumor progression (Subramanian et al., 2007).

### Cell Culture and siRNA-METTL8 Construction

SK-MES-1 and NCI-H520 cells were cultivated in DMEM with 10% fetal calf serum, 100 U/ml of penicillin, and 100 μg/ml of streptomycin. The cells were cultured at 37°C, in 5% CO_2_.

The METTL8-Homo-692(#1) siRNA sequences were as follows: 5′-GGACCUAUGGAGACUGGAUTT-3′ and 5′-AUCC AGUCUCCAUAGGUCCTT-3′. The METTL8-Homo-764 (#2) siRNA sequences were 5′-GGAAAUAGUGUGUUUC CAATT-3′ and 5′-UUGGAAACACACUAUUUCCTT-3′. The METTL8-Homo-1273 (#3) siRNA sequences were 5′-GCACCGAGUGUG GAUUCAATT-3′ and 5′-UUGAAUCCACACUCGGUG CTT-3′. The CTRL siRNA forward: 5′-CCUACGCCACCAAUUU CGU-3′; CTRL siRNA reverse: 5′-ACGAAAUUGGUGGC GUAGG-3′. These oligonucleotides were synthesized by HonorGene (Changsha, China). METTL8 expression was stably knocked down in NCI-H520 cells using the shRNA lentivirus plasmid pLVX-METTL8-sh that targets the human METTL8 gene, which was purchased from HonorGene (Changsha, China) (the target sequence was 5′-GGACCTATGGAGACTGGAT-3′; 5′-GGAAATAGTGTGTTTCCAA-3′ and 5′-GCACCGAGTGTG GATTCAA-3′, respectively). The corresponding vector was pLVX-Puromycin.

### Clinical Samples

A total of 30 LSCC tissues, 20 paracarcinoma tissues, and 16 normal lung tissues were surgically resected in the Second Affiliated Hospital, University of South China (Hengyang, Hunan, China) from 2015 to 2020. The collection and use of tissues were performed in keeping with the ethics standards as formulated in the Declaration of Helsinki. Written informed consent was obtained from each patient, and this protocol was approved by the research ethics committee of the University of South China.

### Immunohistochemistry Staining

Immunohistochemistry (IHC) was performed with a two-step detection kit (Zsbio PV73 9000, China). The paraffin-embedded tissue sections were dewaxed in xylene, rehydrated in a graded alcohol system, and boiled in high-pressure autoclaved citric acid buffer (pH 6.0) for 15 min, and the peroxidase activity was quenched with 3% hydrogen peroxide for 20 min to avoid non-specific staining. The sections were washed three times with PBS followed by incubation overnight with anti-METTL8 antibody (Abcam, ab177201 at 1/200 dilution), PCNA antibody (Abcam, ab265609 at 1/200 dilution), C-myc antibody (Abcam, ab39688 at 1/100 dilution), or Cdc25C antibody (Abcam, ab32444 at 1/200 dilution) at 4°C. After that step, the sections were washed with PBS three times and incubated at room temperature for approximately 20 min with a reaction enhancer kit. This step was followed by three washes in PBS, incubation with secondary antibody at room temperature for 20 min, and staining with 3,3-diaminobenzidine (DAB; Zhongshan Biotech, Beijing, China). The sections were dehydrated and sealed after redyeing with hematoxylin.

### RNA Extraction and qRT-PCR

RNA extraction and PCR progression were performed as previously published, according to manufacturer’s recommendations (Zeng J. et al., 2020). Total RNA was extracted from cells by using TRIzol reagent (Gibco BRL, Grand Island, NY, United States). The PrimeScript RT reagent kit (Perfect Real Time) (Takara Biotechnology Ltd., Dalian, Liaoning, China) was used for the reverse transcription of total RNA to cDNA, which was then stored at −20°C. Glyceraldehyde-3-phosphate dehydrogenase (GAPDH) was selected as an internal reference. RT-qPCR assays were carried out using an ABI7500 qPCR instrument (ABI Company, Oyster Bay, NY, United States). The primer set for METTL8 was F: 5′-GAACACAACATGTGGGA TCA-3′ and R: 5′-CAGCCAATTACGATCCTTG-3′. The primer set for GAPDH was F: 5′-TGGTGAAGGTCGGTGTGAAC-3′ and R: 5′-GGTGGTGAAGACGCCAGTAG-3′. The relative expression level was analyzed using the 2^–ΔΔCt^ formula (Zeng J. et al., 2020).

### Western Blot Analysis

Western blotting was also performed as previously published, according to manufacturer’s recommendations (Zeng J. et al., 2020). Cells were homogenized and sonicated in RIPA buffer (Sigma-Aldrich; Merck KGaA) on ice. Western blotting was conducted according to our previous report. Determination of the protein content: A small amount of the supernatant was collected with a pipette, and the absorbance was measured by a visible spectrophotometer at a wavelength of 590 nm, according to the Bradford method. Using solvent as the blank control and bovine serum albumin (BSA) as the standard control, a curve was drawn, and the contents of the proteins in the extracted samples were estimated based on the standard curve. The extracted proteins were collected, denatured, and electrophoresed through a 10% SDS-polyacrylamide gel. The samples were loaded, and electrophoresis was performed for 60–90 min followed by transfer to PVDF membranes and blocking in 5% skimmed milk at 37°C. After shaking for 2 h, elution was performed. Next, the membranes were incubated with primary antibodies (METTL8, Abcam, Cat. No. ab177201, 1:1,000 dilution; PCNA, Abcam, Cat. No. ab265609, 1:1,000 dilution; c-myc, Abcam, Cat. No. ab39688, 1:1,000 dilution; Cdc25C, Abcam, Cat. No. ab32444, 1:1,000 dilution) at 4°C overnight with shaking. The membranes were then incubated in secondary antibodies (conjugated goat anti-rabbit IgG; CWBIO, Cat. No. CW0103S, 1:2,000 dilution) at room temperature for 2 h and washed in TBST three times for 15 min. Western blot analysis was performed by a chemiluminescence method. The membranes were then incubated in Super Signal ECL-HRP detection reagent (ComWin Biotech) for 1 min followed by exposure to film in a visualizer (Zeng J. et al., 2020).

### Cell Proliferation and Cell Cycle Analysis

Cellular proliferation and cell cycle progression were evaluated by MTT, EdU, and FACS analyses, respectively. All the above methods were conducted following previous reports (Wang et al., 2019; Zeng J. et al., 2020). For the MTT assay, a total of 5 × 10^3^ cells/well were seeded into 96-well plates and cultured for 1, 2, and 3 days at 37°C. Twenty microliters of MTT solution (5 mg/ml, Sigma-Aldrich; Merck KGaA) was incubated for 4 h at 37°C. Then, 150 μl of DMSO was added to dissolve the precipitates, and the effect of cell number on absorbance at 490 nm was measured using a microplate reader (Molecular Devices, LLC). For the EdU assay, cell proliferation was evaluated with an EdU kit (RiboBio, Guangzhou, China). All the assays were repeated three times. For cell cycle analysis, the cells were fixed in ice-cold 70% ethanol and stained with propidium iodide (PI). The cell cycle profiles were assayed using Elite ESP flow cytometry at 488 nm, and the data were analyzed using CELL Quest software (BD Biosciences, San Jose, CA, United States).

### Xenograft Model Antitumor Assay

NCI-H520 cells transfected with the METTL8 shRNA lentivirus plasmid or untransfected were injected into the subcutis of athymic BALB/c nude mice (4 weeks old). Tumor volume (cm^3^) was assessed every 7 days and calculated using a standard formula (width^2^ × length × 0.5). Average tumor volumes were assessed (*n* = 5 for each group) from the seventh day to sacrifice at 70 days. Subsequently, the xenografts were removed, and the tumor sizes and weights were measured at 70 days. The tumor tissues were then fixed in formalin and embedded in paraffin. Afterward, 5-μm thick tissue sections were prepared for subsequent IHC analysis. All experiments were performed according to the guidelines for animal use of the Ethics Committee of the University of South China (Zeng Y. et al., 2020).

### Statistical Analysis

All statistical analyses were performed in the R language (Version 3.6). All statistical tests were bilateral, and *P* < 0.05 was statistically significant.

## Results

### Gene Co-expression Network of Lung Squamous Cell Carcinoma

**FIGURE 1:**
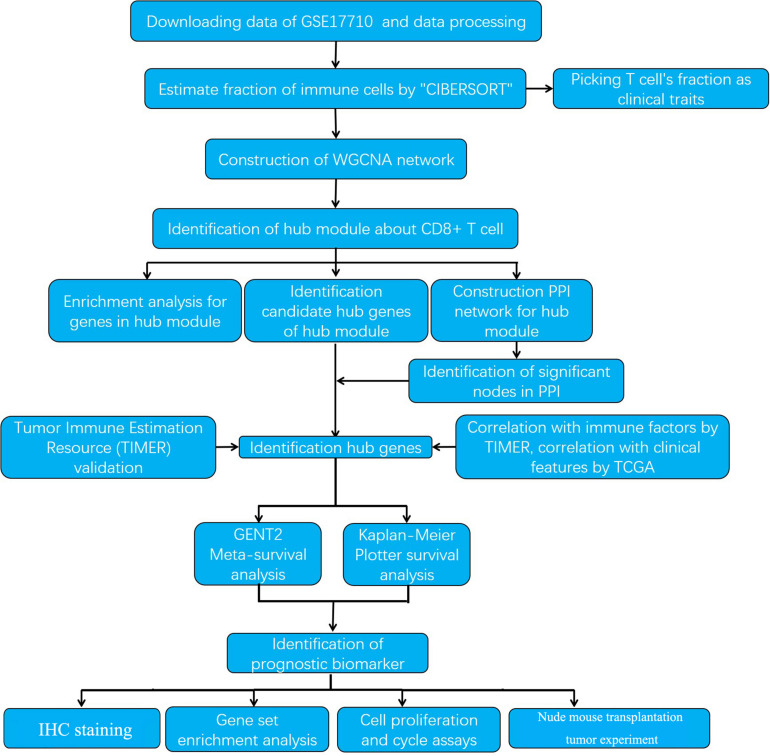
The workflow of the study.

The research strategy is presented in Figure 1.

We extracted RNA level profiles for 56 LSCC samples from Gene Expression Omnibus (GEO) database. All data of the top 5,000 genes with coefficient of variation values greater than 0.1 were selected for additional analysis in this dataset. Subsequently, we used CIBERSORT algorithm to analyze the different cell subtypes abundance for these LSCC samples to calculate the fractions of 22 tumor-infiltrating immune cells (TIICs), in which seven subtypes of T cell fractions were defined as trait data for WGCNA analysis.

**FIGURE 2:**
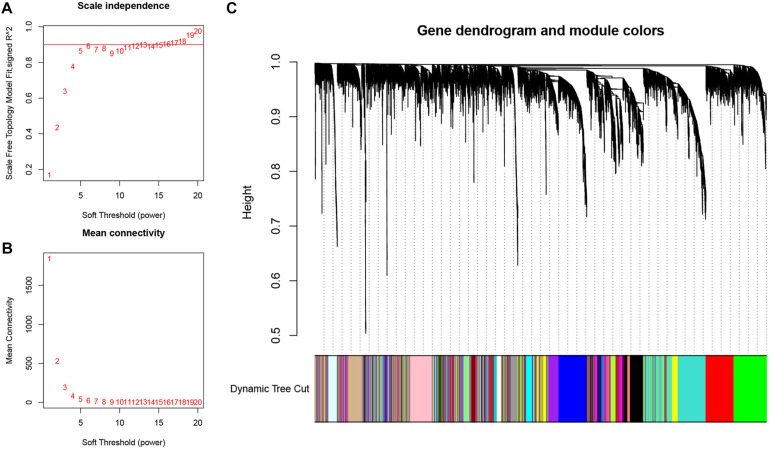
Weighted gene co-expression network analysis (WGCNA) for construction and validation of the hub module. **(A)** The scale-free fit index of the 1–20 soft threshold power (β) is analyzed. **(B)** The average connectivity of 1–20 soft threshold power is analyzed. **(C)** Hierarchical clustering tree of genes based on topological overlap. Different color branches of the cluster tree represent different modules.

The expression profiles of the top 5,000 genes were utilized to construct the gene co-expression network of LSCC by WGCNA analysis. The samples of GSE17710 were clustered by the average linkage and Pearson’s correlation values. We selected β = 5 (scale free *R*^2^ = 0.9) to construct a scale-free network (Figures 2A,B). Then, we utilized dynamic hybrid cutting to build a hierarchical clustering tree, which conducted a gene module. The tree branch represented a series of genes with similar expression data. Each tree leaf represented a single gene on the tree. Furthermore, e18 modules were constructed (Figure 2C).

### Identification of Hub Modules and Enrichment Analysis

**FIGURE 3:**
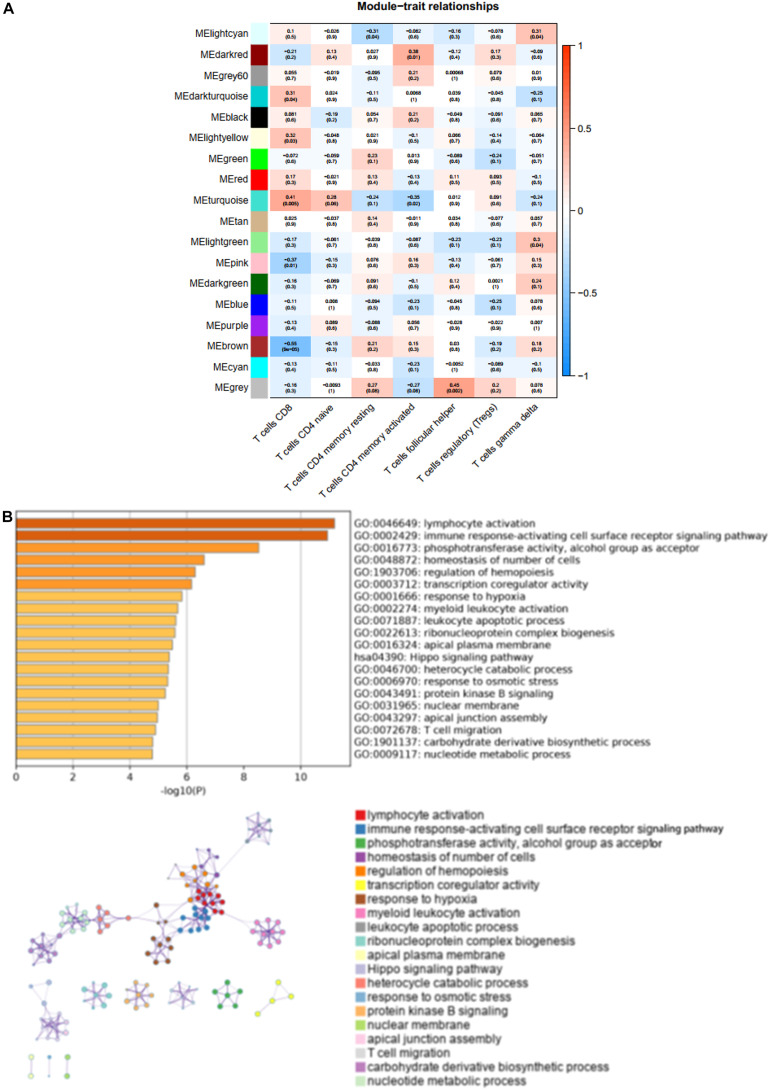
The characteristic notes of hub modules. **(A)** The association between these modules and T-cell infiltration is exhibited by Heatmaps. **(B)** The top 20 enriched terms are shown on the bar chart, and the network of these enriched terms is constructed.

We found that the brown modules were markedly associated to T cells, such as CD8 (CD8^+^ T cells) (*R*^2^ = −0.05, *P* = 9e-05) (Figure 3A). To elucidate the potential function and mechanism of CD8^+^ T cells, we picked the brown modules that exhibited the highest correlation with CD8^+^ T cells, which was validated as a hub module. These genes from the hub brown modules were analyzed by the web tool Matascape for GO and KEGG enrichment analysis, which were mainly enriched in lymphocyte activation, immune response-activating cell surface receptor signaling pathway, and T cell migration (Figure 3B).

### Identification and Validation of Hub Genes

**FIGURE 4:**
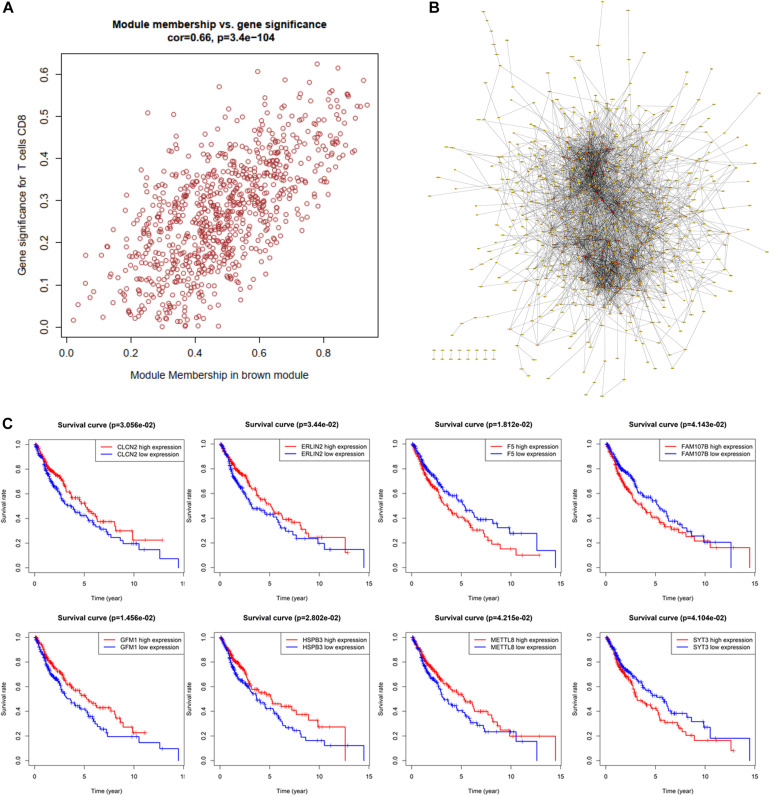
Identification of significant genes related to prognosis. **(A)** All of the genes in the brown module. **(B)** Protein–protein interaction (PPI) network for the brown module. **(C)** Survival analysis is used to screen hub genes based on the TCGA database.

**FIGURE 5:**
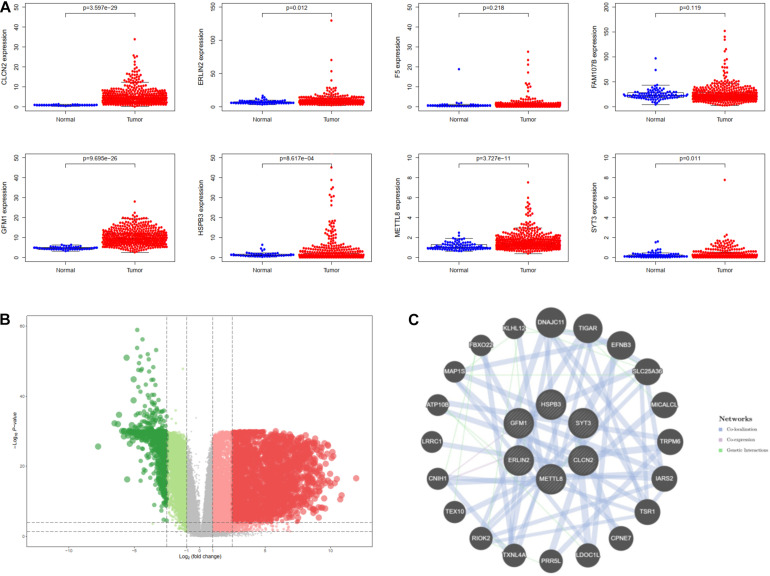
Identification of hub genes for lung squamous cell carcinoma (LSCC). **(A)** The differential expression of these genes in the TCGA database. **(B)** Differentially expressed genes are shown on the volcano plot. **(C)** The interaction of hub six genes based on GeneMANIA database.

For investigating potential hub genes related to CD8^+^ T cell infiltration in the hub modules (Figure 4A), we constructed the protein–protein interactions (PPI) network [cutoff standard: reliability > 0.7 and connectivity > 15 (node/edge)] to identify all of the genes in the hub modules as central nodes, which were visualized by Cytoscape (Figure 4B). Furthermore, we did survival analyses of all the genes in the hub modules, which indicated that eight genes (CLCN2, ERLIN2, F5, FAM107B, GFM1, HSPB3, METTL8, and SYT3) were correlated with the prognosis of LSCC patients based on the TCGA database (Figure 4C). Then, we extracted expression profiles among these eight genes, and we found that six genes (GFM1, HSPB3, SYT3, ERLIN2, METTL8, and CLCN2) have a significant level in LSCC tissues compared with normal lung tissues, respectively (Figure 5A). Volcanic map also indicated that the level of the six genes in LSCC tissues was higher than that in normal tissues (Figure 5B), with corrected p values of all genes less than 0.05, suggesting statistical significance. Moreover, GeneMANIA^5^ indicated that the physical interactions among the six genes were significant in this network (Figure 5C). Therefore, the six genes were selected in both analyses designated as hub genes, to elucidate the relationship between these hub genes and CD8^+^ T cells.

### Determination of Immune and Clinical Characteristics

**FIGURE 6:**
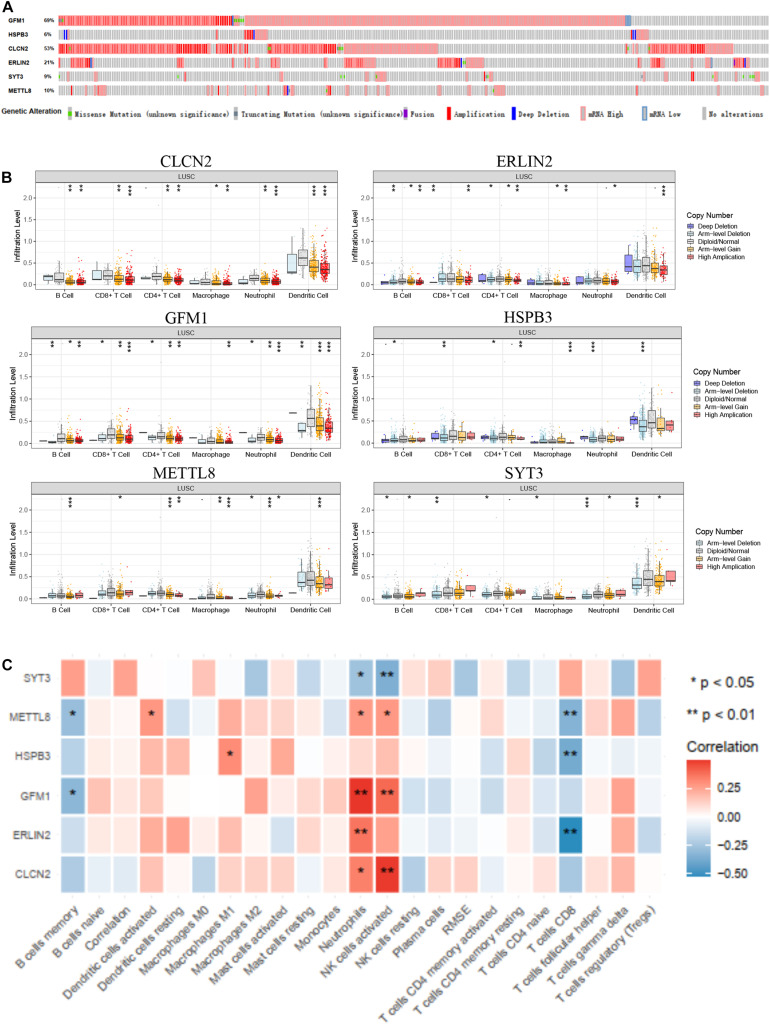
Immune and clinical characteristics of these hub genes. **(A)** The alteration of these hub genes based on cBioPortal database. **(B)** The immune characteristics of these hub genes in the TIMER database. **(C)** The expression profiles of hub genes in multiple immune cell types based on TCGA database.

Owing to the significant immune and clinical characteristics of these hub genes, we made a comprehensive identification of their molecular features. The alteration frequency of GFM1, HSPB3, CLCN2, ERLIN2, SYT3, and METTL8 were 69, 6, 53, 21, 9, and 10% based on cBioPortal database (Figure 6A). Then, we found that amplification and mRNA high were the two most frequently occurring types of mutation. For elucidating the relationship between CD8^+^ T cells and the six hub genes, we extracted the expression level of these hub genes based on the TIMER database, which showed different copy numbers in different immune cell types (Figure 6B). Moreover, we found the correlation of the expression profiles of the three genes with the infiltration levels of CD8^+^ T cells, such as METTL8, HSPB3, and ERLIN2 (Figure 6C). These results suggested that the three hub genes are strongly correlated with the CD8^+^ T cell infiltration and involved in the immune microenvironment.

### Identification of Prognostic Biomarker

**FIGURE 7:**
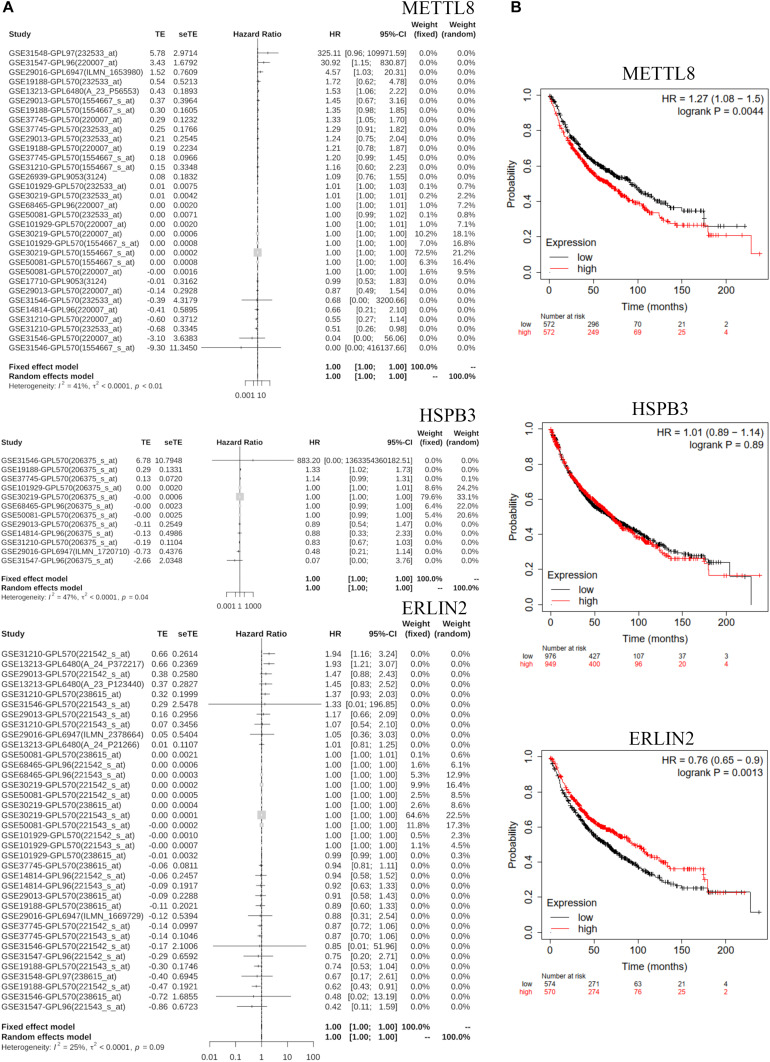
GENT2 Meta-survival analysis and Kaplan–Meier Plotter survival analysis. **(A)** The meta-survival analysis for METTL8, HSPB3, and ERLIN2 based on the GENT2 database. **(B)** The survival analysis of METTL8, HSPB3, and ERLIN2 based on the Kaplan–Meier Plotter database.

We analyzed three hub genes, METTL8, HSPB3, and ERLIN2, by Meta-survival analysis based on GENT2 database^6^. The results showed that the prognostic value of METTL8 and HSPB3 was significantly available for lung cancer patients (Figure 7A). Some of the top datasets showed that the hazard ratios are over one for the same gene and the same tissue region (lung), while others showed that the hazard ratios are under 1. This result suggests that the prognostic value of METTL8 and HSPB3 can be different according to different contexts. We also detected the prognostic value of METTL8, HSPB3, and ERLIN2 in Kaplan–Meier Plotter database^7^. The results indicated that METTL8 and ERLIN2 were significantly and negatively correlated with the prognosis of lung cancer patients (Figure 7B). Taken together, we selected METTL8 as a prognostic biomarker for further analysis.

### Gene Set Enrichment Analysis of METTL8

**FIGURE 8:**
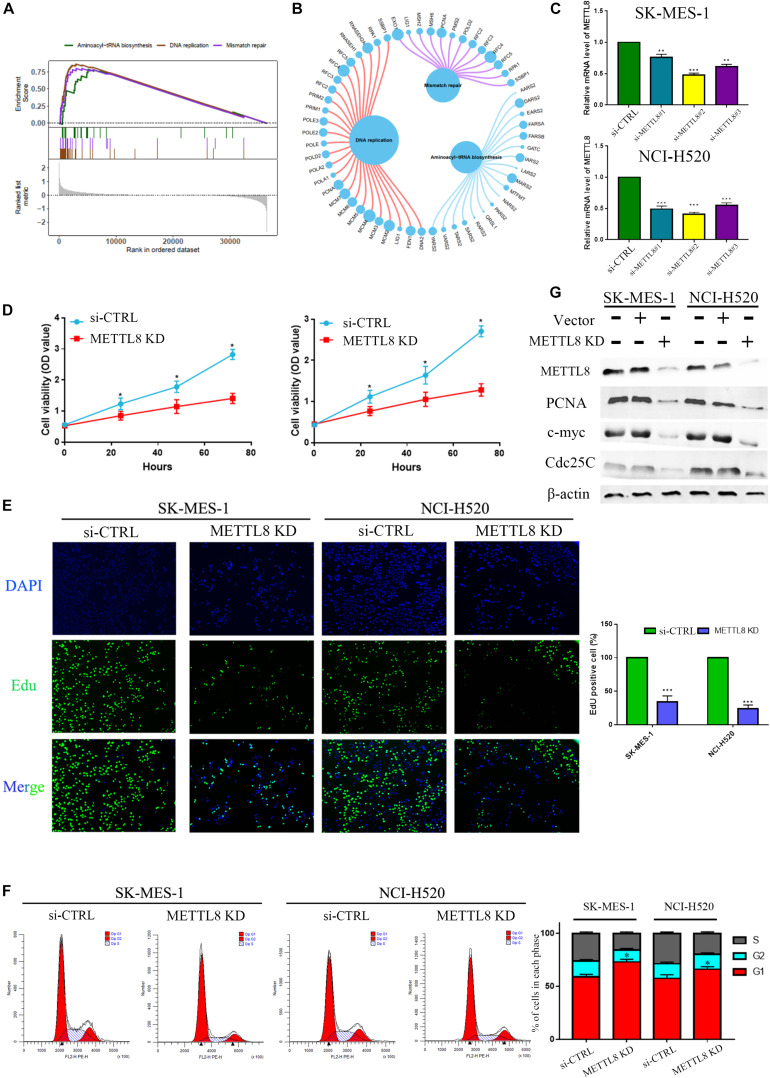
Gene set enrichment analysis (GSEA) and experiment of METTL8. **(A)** The top diagram shows the enriched split lines of the three pathways, and the bottom diagram shows the genes for each pathway. **(B)** The circle shows three enrichment pathways and the significant genes that are involved in the enrichment process. The larger the circle for each gene, the greater the rating scale score. **(C)** The mRNA levels between normal control and si-METTL8 by METTL8 siRNA of SK-MES-1 cells. **(D)** The proliferation ability of si-METTL8 by METTL8 siRNA compared with the normal control by MTT analysis. **(E)** The effect of proliferation of METTL8 by EdU analysis between METTL8 siRNA and NC. **(F)** Cell cycle of si-METTL8 by METTL8 siRNA compared with the normal control by flow cytometry. **(G)** The level of PCNA, c-myc, and Cdc25c of METTL8 KD by shRNA compared with the normal control and vector group by WB.

According to the median value of METTL8 level, LSCC profiles based on the TCGA database were separated into the high-level group and low-level group for GSEA analysis, which indicated that molecular pathways were significantly enriched in the high-level group, with a number of 23 pathways markedly enriched. The top three enriched pathways were Aminoacyl-tRNA biosynthesis, DNA replication, and mismatch repair (Figure 8A). There were no statistically and markedly enriched pathways in the low-level group. Furthermore, three enrichment pathways and the corresponding core gene group that involved in the enrichment progression are displayed in Figure 8B.

### METTL8 Inhibition Significantly Repressed Cell Proliferation and Decelerated Cell Cycle

Owing to the high level of METTL8 in LSCC and its negative correlation with LSCC patient’s prognosis, we hence performed functional experiments to elaborate the possible pathophysiological function of METTL8. At first, we utilized small interfering RNAs to inhibit the METTL8 expression in the LSCC cell line SK-MES-1 (Figure 8C). MTT and EdU analysis indicated that METTL8 knockdown could inhibit the proliferation ability in LSCC cells (Figures 8D,E). Last but not the least, **a** cell cycle analysis was utilized by FACS analysis to clarify the underlying biological function of METTL8-mediated cell cycle acceleration. The result showed that METTL8 knockdown could significantly decelerate the cell cycle (Figure 8F). Moreover, we also found that the expression of PCNA, c-myc, and Cdc25c were obviously decreased in METTL8 inhibition compared with the negative control (NC) and vector group (Figure 8G).

### METTL8 Significantly Promotes Tumor Growth and Is Overexpressed in Lung Squamous Cell Carcinoma Patients

**FIGURE 9:**
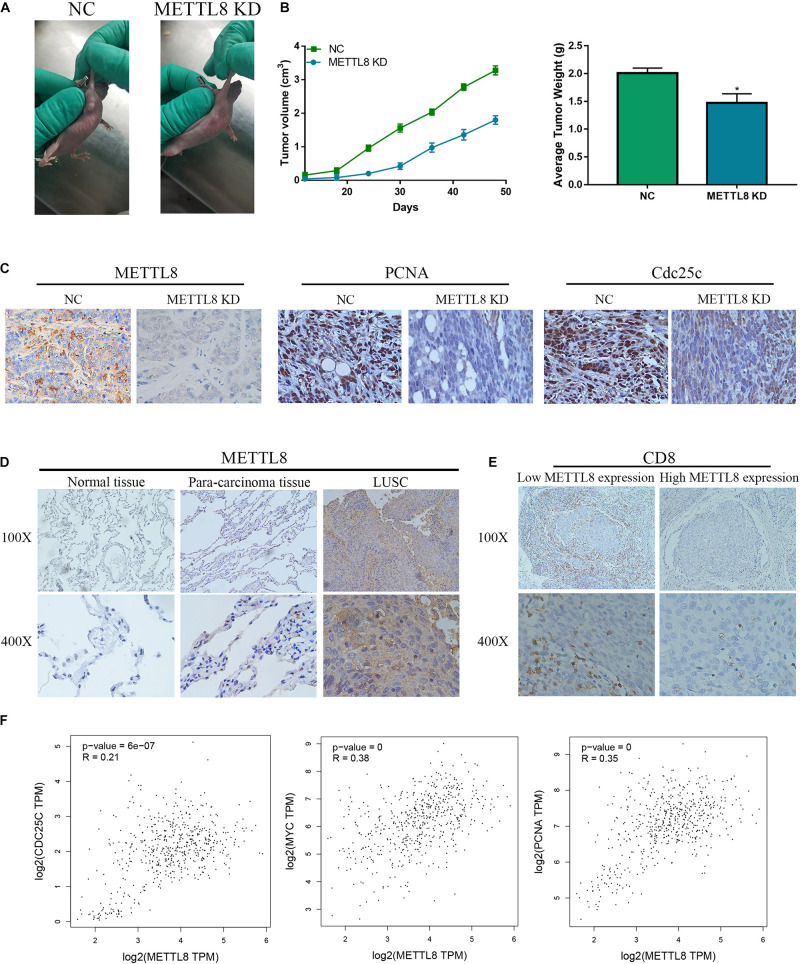
The function and expression of METTL8 *in vivo* and patients. **(A)** NCI-H520 cells with the METTL8 shRNA lentivirus plasmid transfected or untransfected were injected into the subcutis of athymic BALB/c nude mice (4 weeks old). The xenografts were removed and measured at 70 days. **(B)** The tumor volume and weights of the xenografts. **(C)** Immunohistochemetry (IHC) staining was utilized to confirm the level of PCNA and Cdc25c *in vivo*. **(D)** IHC staining was utilized to confirm the level of METTL8 in tissues of normal lung, para-carcinoma, and LSCC. **(E)** The correlation analysis among METTL8, PCNA, myc, and Cdc25c in LSCC based on the TCGA database.

To further explore the role of METTL8 *in vivo*, we used the METTL8 shRNA lentivirus plasmid to inhibit the METTL8 expression in mouse xenograft. We found that the METTL8 KD inhibits the growth of NCI-H520 cells compared with that in the NC group (Figure 9A). Moreover, the tumor sizes and weights in the METTL8 KD group were obviously decreased compared with those in the NC (Figure 9B). IHC staining showed that METTL8 KD inhibited the expression of the proliferation marker PCNA and c-myc, and the cell cycle marker Cdc25c (Figure 9C). We also detected the METTL8 level in normal lung tissues, para-carcinoma tissues, and LSCC tissues, which indicated that the METTL8 level was obviously increased in LSCC compared with that in the normal lung tissues and para-carcinoma tissues (Figure 9D). Furthermore, the expression of METTL8 was positively correlated with PCNA, c-myc, and Cdc25c in LSCC patients based on TCGA database (Figure 9E). Taken together, these results indicated that METTL8 expression was enhanced in LSCC, which could promote tumor growth dependent on PCNA, c-myc, and Cdc25c.

## Discussion

Lung squamous cell carcinoma is one of the most common diseases in the world (Molina et al., 2008). Immune checkpoint inhibitors have shown powerful potential for therapeutic interference with molecular function and signaling in many cancer types and immune cells (Julie et al., 2015; Fehrenbacher et al., 2016; Herbst et al., 2016; Luis et al., 2018). CD8^+^ T cells specific for neoantigen-presenting cancer cells play a significant role in cancer immunotherapy (Daniel and Ira, 2013). In this study, we found that six hub genes were closely correlated with the CD8^+^ T cell infiltration level, indicating a possible mechanism by which six hub genes accelerate the formation, development, and progression of LSCC. Of the 10 identified genes, METTL8 was verified as a potential and significant prognostic marker and target in LSCC. Although different (neo)adjuvant strategies are being developed, anticancer treatment is insufficient to increase the survival rate in LSCC patients. Therefore, immunotherapy requires further exploration, and the complexity of the LSCC microenvironment requires further analysis and larger datasets. We used gene expression matrix data to construct the coexpression network for comprehensive analysis of CD8^+^ T cell infiltration and tested for association between CD8^+^ T cells and the most relevant genes. WGCNA indicated that the brown module was enriched for genes expressed during CD8^+^ T cell infiltration. The most highly associated genes in the coexpression network were significantly correlated with better prognosis, and genes with significantly differential expression were considered hub genes (GFM1, HSPB3, SYT3, ERLIN2, METTL8, and CLCN2). We utilized the TIMER database to elucidate the relationship between six hub genes and CD8^+^ T cell infiltration, which indicated that METTL8, HSPB3, and ERLIN2 were significantly and negatively correlated with CD8^+^ T cells. Furthermore, we used the GENT2 database and Kaplan–Meier Plotter database to ensure that the most important prognostic biomarker among these three genes correlated with CD8^+^ T cells. In the combined meta-survival analysis and overall survival analysis, METTL8 was selected as the most important marker for the early diagnosis and prediction prognosis of LSCC.

Moreover, a study indicated that methyltransferase-like 8 (METTL8) belongs to the methyltransferase-like protein family, which acts mainly to form three-stranded DNA–DNA:RNA hybrid structures to modify m3C in RNA (Xu et al., 2017). Zhang et al. (2020) suggested that METTL8 could regulate R-loops to promote carcinogenesis by nucleic acid base modifications, and they stated that METTL8 could disrupt circular RNA production in their unpublished data. SUMOylated METTL8 induces R-loop and tumorigenesis *via* m3C (Zhang et al., 2020). Ignatova et al. (2019) found that many METTL proteins, such as METTL7B, METTL8, and METTL9, have high-affinity binding partners, resulting in functions outside of stable complexes in HeLa cells. Tobi et al. (2018) indicated that METTL8 might play a key role in adipogenesis to affect lipid metabolism. However, molecular biological function analyses of METTL8 in cancer are limited in colon cancer (Yeon et al., 2018). This study aimed to delineate the biological role of METTL8 in LSCC based on our bioinformatics analysis. Our findings are consistent with those from previous studies on METTL8 (Xu et al., 2017; Yeon et al., 2018). Moreover, multiple LSCC databases suggested that METTL8 was significantly enhanced in LSCC tissues (Wilkerson et al., 2010) and positively correlated with DNA replication, mismatch repair, and aminoacyl-tRNA biosynthesis (Subramanian et al., 2007) in LSCC progression, which indicated that METTL8 may be an oncogene for LSCC. Based on the above bioinformatics analysis and previous literature reports, we further demonstrated the potential role of METTL8 during cancer progression in LSCC. Furthermore, our study also indicated that METTL8 knockdown could reduce the proliferation ability and decelerate the cell cycle *in vivo* and *in vitro*. METTL8, as an M6-adenosine-methyltransferase, can promote the m6A methylation in many cell types (Xu et al., 2017). METTL8 KD may reduce m6A methylation for some mRNAs, miRNAs, or lncRNAs, which can ultimately suppress the proliferation ability and arrest the cell cycle in LSCC cells. Moreover, these non-coding RNAs methylated by METTL8 may be delivered to CD8^+^ T cells *via* exosomes, which may influence CD8^+^ T cell immune infiltration in LSCC (Li et al., 2016). However, the specific molecular mechanism remains unclear.

Our study also indicated that METTL8 plays a key role in the immune infiltration of LSCC tumors. The CIBERSORT algorithms showed that overexpression of METTL8 was negatively correlated with CD8^+^ T cell immune infiltration, and the function of METTL8 was enriched in lymphocyte activation, immune response-activating cell surface receptor signaling pathway, and T cell migration. All the WGCNA, GO, KEGG, and CIBERSORT algorithms indicated that METTL8 expression was markedly negatively associated with CD8^+^ T cell. Furthermore, the experiment also demonstrated that METTL8 was negatively associated with CD8^+^ T cells. IHC staining also indicated an obvious and negative correlation between METTL8 expression and CD8^+^ T cell infiltration in LSCC patients. Moreover, this study is the first to reveal the correlation between METTL8 overexpression and CD8^+^ T cell immune infiltration in LSCC.

In the past few years, CD8^+^ T cell immune infiltration can be used as an index to early diagnose LSCC (Jiang et al., 2019), indicating that CD8^+^ T cell immune infiltration has great potential in personalized immunotherapy trials for LSCC (Trojan et al., 2004). We found that METTL8 has an obvious and negative association between CD8^+^ T cell immune infiltration based on bioinformatic analysis and IHC staining. However, T cells contain a mixture of T cell subtypes, including regulatory T cells (Treg cells), CD4^+^, and CD8^+^ cells (Hao et al., 2020). These diverse T cell types have different molecular functions, and the function of METTL8 in the biological progression of different T cell types remains unclarified. Therefore, exploring the role of METTL8 in T cells is one of the main directions in the future for us. FOXP3, a transcription factor, is overexpressed in Treg cells and is utilized to distinguish Treg cells (CD4^+^ FOXP3^+^) (Kim, 2009; Hu et al., 2019). The main biological function of Treg cells is to suppress immune activity, but their pathophysiological significance in LSCC is still unclear (Yang et al., 2017; Hao et al., 2020). Because of database limitations, the correlation between METTL8 and FOXP3 is still unclear in T cells. Furthermore, the relationship between METTL8 and FOXP3 may be a main role in tumor immune escape for LSCC patients. Moreover, CD8^+^ cells with immunosuppressive effects were found by Maoz and Cantor in 1971 (Cantor et al., 1976; Maoz et al., 1976). The other Treg cells (CD8^+^ FOXP3^+^) only account for a small group of Treg cells. Reportedly, Treg cells (CD8^+^ FOXP3^+^) have a neglected but strong suppressive effect on the immune system in colon cancer (Chaput et al., 2009), but the function of Treg cells (CD8^+^ FOXP3^+^) in LSCC is less widely studied (Hao et al., 2020). In our results, we found the negative correlation between METTL8 and CD8^+^ T cell immune infiltration based on bioinformatic and experimental analysis, but it is still unclear whether these CD8 cells belong to which subtype. Therefore, the association between METTL8 and FOXP3 needs to be improved urgently in T cell.

To the best of our knowledge, this study is the first to explore the correlation between METTL8 expression and CD8^+^ T cell immune infiltration in LSCC. Nevertheless, there are some limitations. First, the conclusion of our study was based on public databases, which are influenced by the data quality. Subsequently, the biological function of METTL8 in LSCC progression was only preliminarily demonstrated by proliferation assays and some immune assays with LSCC patient tissues. Although the regulation of CD8^+^ T cell immune infiltration by METTL8 is unclear, the results are promising and worth further study.

In summary, our study indicated that a high level of METTL8 induced LSCC progression and was negatively associated with CD8^+^ T cell immune infiltration. Therefore, we found that METTL8 might be a novel prognostic biomarker and an immunotherapeutic target in LSCC.

## Data Availability Statement

All the gene expression data were extracted from public databases, including GEO, TCGA, GDSC, and Genemania database. The GSE66957 (https://www.ncbi.nlm.nih.gov/geo/query/acc.cgi?acc=GSE66957) and GSE54388 (https://www.ncbi.nlm.nih.gov/geo/query/acc.cgi?acc=GSE54388) dataset were extracted from GEO database.

## Ethics Statement

The studies involving human participants were reviewed and approved by University of South China. The patients/participants provided their written informed consent to participate in this study.

## Author Contributions

MT, S-JT, JH, and YL conceptualized and designed the study. XL, JX, JW, XC, and QH were in charge of the collection and assembly of the data. XZ and YL conducted the data analysis and interpretation. MT, YL, and XL wrote the manuscript. JH and S-jT made the manuscript revision. All authors did the final approval of the manuscript.

## Conflict of Interest

The authors declare that the research was conducted in the absence of any commercial or financial relationships that could be construed as a potential conflict of interest.

## Footnotes

1 https://portal.gdc.cancer.gov/

2 https://www.ncbi.nlm.nih.gov/geo/

3 www.metascape.org

4 https://cistrome.shinyapps.io/timer/

5 http://genemania.org/

6 http://gent2.appex.kr

7 http://kmplot.com/
